# Practices Towards Energy Drink Consumption Among the Students of a Medical College in New Delhi, India

**DOI:** 10.7759/cureus.79819

**Published:** 2025-02-28

**Authors:** Samar Hossain, Amod Laxmikant Borle, Irena Mandal, Ekta Arora, Anshita Mishra, Titikshaa Gupta, Manpreet Singh, Shivangi Baghel

**Affiliations:** 1 Community Medicine, Maulana Azad Medical College, New Delhi, IND

**Keywords:** caffeine, delhi, energy drinks, medical students, sports drinks

## Abstract

Introduction: The consumption of energy drinks among adolescents and young adults is on the rise, driven by claims of enhanced energy and performance. However, many students consume these beverages without full awareness of their potential effects, making it a public health concern. This study aims to assess the prevalence and consumption patterns of energy drinks among medical college students in Delhi.

Aims and objectives: This study aims to assess the prevalence, patterns, and knowledge of energy drink consumption among medical students in Delhi, while also exploring the factors that influence their consumption behavior. It seeks to determine how commonly undergraduate medical students consume energy drinks and to analyze their consumption patterns, including the frequency of intake, age of initiation, and motivations behind their use. Additionally, the research evaluates students' knowledge and awareness regarding the ingredients of energy drinks, their differences from sports drinks, and the potential harmful effects associated with their consumption. Furthermore, the study investigates the socio-demographic and lifestyle factors, such as gender, year of study, and leisure screen time, that may be linked to energy drink consumption and the level of knowledge among medical students.

Methods: A cross-sectional study was conducted among 226 undergraduate students at Maulana Azad Medical College, New Delhi. Prior to the study, ethical approval was obtained from the Institute Ethics Committee, and permission was secured from the Dean. Participants were selected through simple random sampling from a list provided by the Dean’s office. After obtaining informed consent, the investigator conducted face-to-face interviews using a self-designed, pre-tested questionnaire covering socio-demographic details, knowledge of energy drinks, and consumption practices. Statistical analysis was performed using the Chi-square test to examine associations between variables, with a p-value ≤0.05 considered statistically significant.

Results: Among the 226 participants, the majority (87.6%) were in the 19-24 years age group. Energy drink consumption was reported by 62% of participants, with 75% of consumers being occasional users (less than one drink per week). Over half of the consumers (60.4%) were aware of the harmful effects of energy drinks, while 59.7% had knowledge of their ingredients. Gender, year of study, and leisure screen time showed significant associations with multiple knowledge-related variables.

Conclusion: More than 50% of participants were identified as energy drink consumers. A significant association was found between gender, year of study, and preferred energy drink brand with knowledge and consumption patterns related to energy drinks.

## Introduction

People for centuries have tried to find ways to stay energized but when coffee failed, energy drinks prevailed. Energy drinks are non-alcoholic beverages that contain high levels of caffeine [1-3], sugar, and amino acid taurine [4]. Most energy drinks contain 80-150 mg of caffeine per serving similar to a cup of coffee [5]. Caffeine is a stimulant and in high amounts causes increased attention span, enhanced memory, and better mood [6]. Studies have even shown a negative association between cognitive decline and caffeine intake [7]. These beneficial effects are often highlighted through overenthusiastic marketing by energy drink companies.

Energy drinks have the potential to cause harm due to their ingredients; arrhythmias, anxiety, insomnia, restlessness, caffeine toxicity, gastrointestinal upset, Type 2 diabetes mellitus, and obesity [6]. The need for creating awareness about these effects is paramount as the consumers are the young generation. 

With the rise of popular energy drinks in the Indian market exceeding the recommended daily caffeine intake, the Food Safety and Standards Authority of India (FSSAI) has implemented regulations on caffeinated beverages, capping the maximum permissible caffeine limit at 300 mg per liter [8]. The consumption was earlier limited due to the lack of affordability, however, with cheaper alternatives, the market size of energy drinks is growing by the day, estimated at Rs 2,400 crore, growing at a CAGR (compound annual growth rate) of 45-50% [9].

Energy drinks are often confused with sports drinks that primarily contain electrolytes and carbohydrates and are intended to replenish water and electrolytes lost during the workout. The latter does not contain any stimulants [10].

The consumption of energy drinks by adolescents and young adults is on the rise as they claim to increase energy and performance [1]. There are other reasons to use energy drinks such as to keep awake, to give company to peers, to improve performance in the exams, for the taste, and sometimes, to experiment [2]. This is a cause for concern as they are often unaware of the contents and consume heedlessly, thus making energy drinks a potential public health hazard [11].

The importance of highlighting the attitude of youth toward energy drinks is to increase awareness about the composition of the beverage, its effect on physical and mental health, academic performance, caffeine toxicity, and alcohol dependence [12-14]. The current study was thus undertaken to study the knowledge and practices among the students of a medical school in New Delhi.

## Materials and methods

A cross-sectional study was conducted over a six-month period (March-August 2023) among medical students at Maulana Azad Medical College, New Delhi. The study included all medical students who had been residing in the study area for at least six months, while those who were severely ill or unable to communicate were excluded. The sample size was determined to be 222, based on an energy drink consumption prevalence of 17.5%, a relative allowable error of 5%, and a 95% confidence level [13]. A total of 226 participants were ultimately enrolled in the study.

Before initiating the study, ethical approval was obtained from the Institute Ethics Committee (approval number: F.801(5)/2018/RC/Vol.I/879R), along with formal permission from the Dean of Maulana Azad Medical College, New Delhi. 

For sampling, a list of all undergraduate students was prepared, and participants were selected using a computer-generated random number table, ensuring that each student had an equal probability of selection. Each eligible student was assigned a unique identification number, and selection was conducted through statistical software, minimizing selection bias.

The study employed a simple random sampling method; however, to enhance reproducibility, a detailed description of the randomization process is necessary. Future research should provide step-by-step documentation of the method to improve transparency.

Only those students who provided informed consent were enrolled in the study. The investigator then conducted interviews using a self-designed, pre-tested questionnaire, which included sections on socio-demographic details, knowledge of energy drinks, and consumption practices. However, while the questionnaire was pre-tested, its validity and reliability were not explicitly established before implementation, raising concerns about data accuracy and consistency. Future studies should incorporate pilot testing, expert content validation, and reliability assessments (such as Cronbach’s alpha for internal consistency) to enhance data credibility and reproducibility.

In this study, knowledge assessment was based on a scoring system, where participants were asked multiple-choice and open-ended questions regarding energy drink ingredients, differences from sports drinks, and harmful effects. A participant was considered knowledgeable if they correctly answered at least 70% of the questions in each category. The questionnaire included fact-based statements aligned with scientific literature and regulatory guidelines, ensuring an objective evaluation. However, the specific criteria for categorizing knowledge levels were not explicitly stated, which may affect the consistency of the findings. Future studies should adopt validated scoring frameworks to enhance comparability and reliability in knowledge assessment.

After data collection, responses were entered into Microsoft Excel, followed by data cleaning and processing. Statistical analysis was conducted using SPSS software (version 25). Quantitative variables were summarized as mean and standard deviation (SD), while qualitative variables were expressed as proportions. The chi-square test was applied to assess associations, with a p-value ≤0.05 considered statistically significant.

## Results

A total of 226 participants were included in the study. Table 1 outlines the socio-demographic characteristics and lifestyle factors of the participants. The majority (216, 95.6%) were undergraduate students, with 198 (87.6%) belonging to the 19-24 years age group. Males constituted 57.5% (130) of the sample, and 68 (30.1%) were first-year MBBS students. Nearly all participants (223, 98.7%) were unmarried.Among lifestyle factors, 36.3% reported engaging in physical activity 2-3 times per week, while 31.4% had leisure screen time of 2-3 hours per day. The definition of physical activity used in the questionnaire was based on WHO recommendations for individuals aged 18-64 years, which suggest either 150-300 minutes of moderate aerobic activity or 75-150 minutes of vigorous activity per week (Table 1).

**Table 1:** Socio-demographic and lifestyle factors of the subjects (n=226).

(N=226)		
Variables		Number (%)
Age (in years)	18 and below	16 (7.1)
	19-24	198 (87.6)
	25-29	9 (4.0)
	30 and above	3 (1.3)
Gender	Male	130 (57.5)
	Female	96 (42.5)
Education	MBBS 1st-year	68 (30.1)
	MBBS 2nd-year	47 (20.8)
	MBBS 3rd-year	62 (27.4)
	MBBS 4th-year	32 (14.2)
	MBBS Intern	17 (7.6)
Frequency of physical activity	Daily	65 (28.8)
	2-3 times per week	82 (36.3)
	Once a week	40 (17.7)
	Once a month	16 (7.1)
	Never	23 (10.2)
Leisure screen time (per day)	Less than 2 hours	31 (13.7)
	2-3 hours	71 (31.4)
	3-4 hours	52 (23.0)
	4-6 hours	52 (23.0)
	More than 6 hours	20 (8.8)

**Figure 1:**
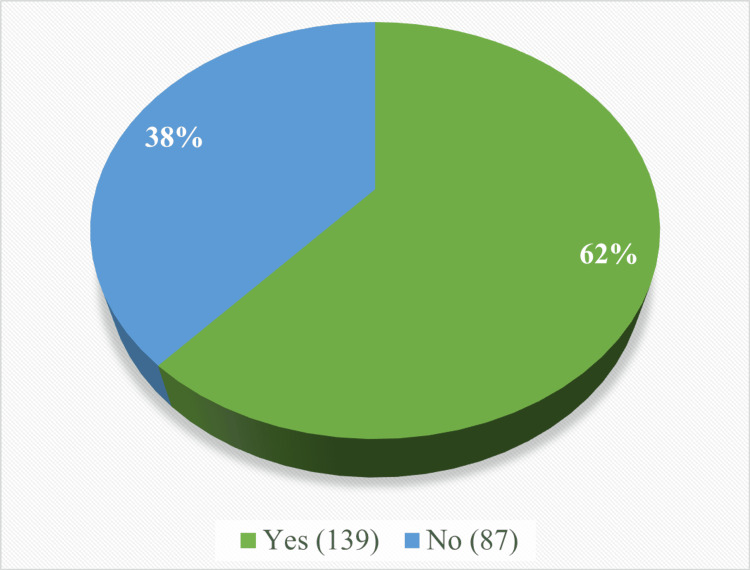
Distribution of study participants according to consumption of energy drinks.

Out of 226 participants, 139 (62%) were identified as energy drink consumers (Figure 1). 

While 83, (59.7%) reported being aware of energy drink ingredients, only 69, (49.6%) could distinguish them from sports drinks. However, the majority (84, 60.4%) were aware of the harmful effects of energy drinks (Table 2).

**Table 2:** Knowledge regarding energy drinks among consumers (N=139).

(N=139)		
Variables	Response	
	Yes (%)	No (%)
Ingredients of energy drinks	83 (59.7)	56 (40.3)
Different from sports drinks	69 (49.6)	70 (50.4)
Harmful effects of energy drinks	84 (60.4)	55 (39.6)

Table 3 presents the statistical associations between knowledge of energy drinks and various socio-demographic and lifestyle factors. Gender and year of study showed a significant association with awareness of the differences between sports drinks and energy drinks, knowledge of energy drink ingredients, and understanding of their long-term harmful effects.

**Table 3:** Association of knowledge regarding energy drinks with various socio-demographic factors and lifestyle factors.

(N=226)							
Variables		Aware that sports drinks are different from energy drinks	Test statistical value, p-value	Aware of the ingredients of energy drinks	Test statistical value, p-value	Aware of the long-term harmful effects of energy drinks	Test statistical value, p-value
		Yes n (%)		Yes n (%)		Yes n (%)	
Gender	Male	45 (65.2)	7.729, 0.02	55 (66.3)	7.910, 0.01	52 (61.9)	8.419, 0.01
	Female	24 (34.8)		28 (33.7)		32 (38.1)	
Year of study	MBBS 1^st^ year	13 (18.8)	20.884, 0.01	17 (20.5)	22.326, 0.007	27 (32.1)	20.072, 0.02
	MBBS 2^nd^ year	16 (23.2)		20 (24.1)		22 (26.2)	
	MBBS 3^rd^ year	22( 31.9)		24 (28.9)		18 (21.4)	
	MBBS 4^th^ year	11 (15.9)		12 (14.5)		8 (9.5)	
	MBBS Intern	7 (10.1)		10 (12)		9 (10.7)	
Leisure screen time	<2 hours	6 (8.7)	24.794, 0.002	7 (8.4)	9.038, 0.362	12 (14.3)	12.457, 0.20
	2-3 hours	27 (39.1)		30 (36.1)		28 (33.3)	
	3-4 hours	13 (18.8)		20 (24.1)		21 (25.0)	
	4-6 hours	22 (31.9)		20 (24.1)		19 (22.6)	
	>6 hours	1 (1.4)		6 (7.2)		4 (4.8)	

Table 4 presents the consumption patterns of energy drinks among the study participants. The majority (105, 75.6%) were occasional consumers, defined as those who consumed energy drinks less than once per week. The most common age of initiation was 16-19 years, reported by 69 (49.6%) participants. In terms of weekly intake, 102 (73.4%) consumed less than one bottle/can per week, while 130 (93.5%) purchased energy drinks from local stores near their hostels or residences. Additionally, 125 (90%) participants did not mix energy drinks with alcohol; however, 4.4% (6) reported combining them with beverages such as beer, vodka, tequila, and wine.

**Table 4:** Practice regarding energy drinks (N=139)

(N=139)		
Variables		Number (%)
Frequency	Daily	9 (6.4)
	2-3 times per week	12 (8.6)
	Once a week	13 (9.4)
	Occasionally	105 (75.6)
Initiation of consumption	Less than 15 years	22 (15.8)
	16-19 years	69 (49.6)
	19-24 years	46 (33.2)
	24-29 years	2 (1.4)
Weekly consumption (in number of bottles/cans)	Less than 1	102 (73.4)
	1-2	22 (15.8)
	3 or more	15 (10.8)
Purchased from	Online	9 (6.5)
	Local store	130 (93.5)
Mixing with alcoholic beverage	Vodka	10 (7.2)
	Beer	2 (1.4)
	Tequila	1 (0.7)
	Wine	1 (0.7)
	None	125 (90)

Table 5 shows the consumption patterns of energy drinks in relation to gender, revealing a significant association with mixing energy drinks with alcohol. Females were found to mix energy drinks with alcohol at twice the rate of males. In terms of frequency of consumption, more males (7, 77.8%) reported daily consumption compared to females (2, 22.2%), though the association was not statistically significant (p=0.32). Similarly, among those consuming energy drinks 2-3 times per week, males constituted 58.3%, while females made up 41.7%. The trend continued with weekly consumption, where 84.6% (11) were male and 15.4% (2) were female. Occasional consumption was reported by 65 males (61.9%) and 40 females (38.1%). A significant gender difference was observed in the practice of mixing energy drinks with alcohol (p=0.033). Among those who engaged in this practice, 35.7% (5) were male, while 64.3% (9) were female. In contrast, among those who did not mix energy drinks with alcohol, the majority were male (81, 64.8%) compared to females (44, 35.2%). Regarding the age of initiation, most participants who started consuming energy drinks before the age of 15 were male (17, 77.3%) compared to females (5, 22.7%). Among those who initiated between 16-19 years, 66.7% (46) were male, and 33.3% (23) were female. In the 19-24-year age group, 56.5% (26) were male and 43.5% (20) were female, while in the 24-29-year group, both genders were equally represented (1, 50%). However, the association between age of initiation and gender was not statistically significant (p=0.365).

**Table 5:** Association of practice regarding energy drinks with gender.

	(N=139)					
Variable			Male N (%)	Female N (%)	Test statistical value	p-value
Frequency of consumption		Daily	7 (77.8)	2 (22.2)	10.991	0.32
		2-3 times per week	7 (58.3)	5 (41.7)		
		Once a week	11 (84.6)	2 (15.4)		
		Occasionally	65 (61.9)	40 (38.1)		
Mixing energy drinks with alcohol		Yes	5 (35.7)	9 (64.3)	7.718	0.033
		No	81 (64.8)	44 (35.2)		
Age of initiation		<15 years	17 (77.3)	5 (22.7)	10.685	0.365
		16-19 years	46 (66.7)	23 (33.3)		
		19-24 years	26 (56.5)	20 (43.5)		
		24-29 years	1 (50)	1 (50)		

## Discussion

A high prevalence (74.88%) of energy drink consumption was reported among medical students higher than our study (62%) with the consumption being higher in male students. The prevalence of energy drink consumption among adolescents in Delhi was reported to be 55% and 52% by Usman et al., which closely aligns with the findings of our study and some more studies [15,16,17]. Consumption was found to be lower compared to a study by Aslam et al., which reported that only 42% of medical students consumed energy drinks [18]. It was reported that in non-medical students, energy drinks are perceived to be beneficial and the frequent consumers were unaware of the ingredients unlike our study [19].

Edrees et al. reported a higher level of knowledge (70.2%) about energy drink ingredients among 21 to 23-year-old medical students, which is comparable to our study's findings (59.3%) [15]. Pangtey et al. and Rahamathulla et al. reported that many adolescents consumed energy drinks due to a lack of awareness about their harmful effects [16,20]. In contrast, our study found that the majority of students were aware of the adverse effects of energy drinks. Our study found a higher level of knowledge regarding the correct definition of energy drinks and their distinction from sports drinks compared to Aslam et al., where both users and non-users exhibited similar levels of awareness [18]. The present study found statistically significant differences in all knowledge-related variables based on gender and year of study, aligning with findings from previous research [21].

In terms of consumption frequency, this study found that most participants were occasional consumers, consistent with findings from previous research [21]. Our study found that energy drink consumption was most commonly initiated between the ages of 16 and 19, a pattern not previously examined. This underscores the importance of focusing health education efforts on this age group to raise awareness about energy drink consumption.

With respect to weekly consumption, our study observed that most participants consumed less than one energy drink per week, likely due to factors such as sharing a bottle with friends. These findings align with those reported in previous studies. In our study, nearly 90% of participants purchased energy drinks from local stores, with most sales occurring at small pan and cigarette shops. This highlights the need for effective regulatory measures to address the availability of these beverages near educational institutions.

Our study found that only 10% of participants consumed energy drinks mixed with alcohol, a trend not previously reported in other studies. This may be due to ethical concerns surrounding alcohol consumption and varying age restrictions across different regions. However, similar proportions of participants in previous studies cited reasons such as "other" or "during parties" for consumption [18]. Additionally, our study identified a significant association between gender and alcohol mixing, with females engaging in this practice at nearly twice the rate of their male counterparts. The highest consumption was observed among participants who reported leisure screen time of 2-4 hours, consistent with the findings by Degirmenci et al. [22].

Strengths of the study

One of the key strengths of this study is its comprehensive assessment of energy drink consumption patterns among medical students, offering insights into knowledge, attitudes, and behaviors. The study also highlights gender-specific and lifestyle-related associations, providing a foundation for targeted public health interventions.

Limitations of the study

While the study effectively outlines its methodology, including sampling procedures and statistical analysis, certain limitations should be acknowledged to enhance transparency and reproducibility. The lack of questionnaire validation raises concerns about data reliability, as details regarding pilot testing, expert validation, or reliability assessments such as Cronbach’s alpha were not provided. Additionally, although simple random sampling was employed, the specific randomization tool used for participant selection was not explicitly described, which may impact reproducibility. Addressing these limitations in future research will strengthen the accuracy, reliability, and credibility of the findings, ensuring better methodological rigor and reproducibility.

## Conclusions

Despite being aware of energy drinks and their harmful effects, a majority of participants were still consumers, highlighting a serious concern that warrants further investigation to understand the underlying behavioral factors driving this consumption. Our findings indicate a strong association between knowledge and gender, academic year, and leisure screen time, suggesting that these variables play a crucial role in shaping awareness and consumption patterns. Additionally, the mixing of energy drinks with alcohol was significantly associated with gender, emphasizing the need for targeted interventions.

The findings emphasize the need for stricter regulations on energy drink marketing and sales, especially near educational institutions. Targeted awareness campaigns focusing on gender-based consumption differences and the risks of mixing energy drinks with alcohol are essential. Given that future healthcare professionals are among the consumers, it is crucial to integrate evidence-based health education programs into medical curricula to address risky consumption behaviors early on. Developing behavioral interventions tailored to specific high-risk groups could contribute to reducing long-term health risks associated with excessive energy drink consumption.
